# Prognostic gene signatures for patient stratification in breast cancer - accuracy, stability and interpretability of gene selection approaches using prior knowledge on protein-protein interactions

**DOI:** 10.1186/1471-2105-13-69

**Published:** 2012-05-01

**Authors:** Yupeng Cun, Holger FH Fröhlich

**Affiliations:** 1 Algorithmic Bioinformatics, Bonn-Aachen International Center for IT, Dahlmannstraße. 2, 53113, Bonn, Germany

## Abstract

**Background:**

Stratification of patients according to their clinical prognosis is a desirable goal in cancer treatment in order to achieve a better personalized medicine. Reliable predictions on the basis of gene signatures could support medical doctors on selecting the right therapeutic strategy. However, during the last years the low reproducibility of many published gene signatures has been criticized. It has been suggested that incorporation of network or pathway information into prognostic biomarker discovery could improve prediction performance. In the meanwhile a large number of different approaches have been suggested for the same purpose.

****Methods**:**

We found that on average incorporation of pathway information or protein interaction data did not significantly enhance prediction performance, but indeed greatly interpretability of gene signatures. Some methods (specifically network-based SVMs) could greatly enhance gene selection stability, but revealed only a comparably low prediction accuracy, whereas Reweighted Recursive Feature Elimination (RRFE) and average pathway expression led to very clearly interpretable signatures. In addition, average pathway expression, together with elastic net SVMs, showed the highest prediction performance here.

****Results**:**

The results indicated that no single algorithm to perform best with respect to all three categories in our study. Incorporating network of prior knowledge into gene selection methods in general did not significantly improve classification accuracy, but greatly interpretability of gene signatures compared to classical algorithms.

## Background

Molecular biomarkers play an important role in clinical genomics. Identification and validation of molecular biomarkers for cancer diagnosis, prognosis, and subsequent treatment decision becomes an important problem in personalized medicine. Modern technologies, like DNA microarrays and deep sequencing methods, can measure thousands of gene expression profiles at same time, which can be used to indentify patterns of gene activity that might provide criteria for individual risk assessment in cancer patients.

Biomarker discovery poses a great challenge in bioinformatics due to the very high dimensionality of the data coupled with a typically small sample size. In the past a large number of classification algorithms have been developed or adopted from the machine learning field, like PAM, SVM-RFE, SAM, Lasso and Random Forests [1-4]. Several adaptations of Support Vector Machines (SVM) [5] have been suggested for gene selection in genomic data, like L1-SVMs, SCAD-SVMs and elastic net SVMs [6-8]. Although these methods show reasonably good prediction accuracy, they are often criticized for their lack of gene selection stability and the difficulty to interpret obtained signatures in a biological way [9,10]. These challenges provide opportunities for the development of new gene selection methods.

To overcome the disadvantages of conventional approaches Chuang et al. [11] proposed an algorithm that incorporates of protein-protein interaction information into prognostic biomarker discovery. Since then a number of methods going into the same direction have been published [11-17].

In this article, we compared fourteen published gene selection methods (eight using network knowledge) on six public breast cancer datasets with respect to prediction accuracy, biomarker signature stability and biological interpretability in terms of an enrichment of disease related genes, KEGG pathways and known drug targets. We found that incorporation of network information could generally not improve prediction accuracy significantly, but could sometimes indeed improve gene selection stability and biological interpretability of biomarker signatures drastically. Specifically, Reweight Recursive Feature Elimination (RRFE) [17] and average pathway expression led to a very clear interpretation in terms of enriched disease relevant genes, pathways and drug targets. On the other hand, network-based SVMs [15] yielded the most stable gene signature.

## Methods

### **Gene selection methods**

We employed fourteen published gene selection methods in this article. In machine learning features selection methods can be classified into three categories [18]: filters, wrappers and embedded methods. Filter methods select a subset of features prior to classifier training according to some measure of relevance for class membership, e.g. mutual information [19]. Wrapper methods systematically assess the prediction performance of feature subsets, e.g. recursive feature elimination (RFE) [3]; and embedded methods perform features selection within the process of classifier training. The methods we employed in this article covered all three categories. Furthermore we can classify feature selection methods according to whether or not they incorporate biological network knowledge (conventional vs. network-based approaches).

As one of the most basic approaches, we considered here a combination of significance analysis of microarrays (SAM) [20] as a filter prior to SVM or Naïve Bayes classifier learning. More specifically, only genes with FDR < 5% (Benjamini-Hochberg method) [21] were considered as differentially expressed. As further classical gene selection methods we considered prediction analysis for microarrays (PAM) [2], which is an embedded method, and recursive feature elimination (SVM-RFE) [3], an SVM-based wrapper algorithm. Moreover, we included SCAD-SVMs [7] and elastic-net penalty SVMs (HHSVM) [8] as more recently proposed embedded approaches that particularly take into account correlations in gene expression data. In this article we used SAM+SVM (significant gene SVM), SAM+NB (significant gene Naïve Bayes classifier), PAM, SCAD-SVM, HHSVM and SVM-RFE as conventional feature selection methods that do not employ network knowledge.

The following network-based approaches for integrating network or pathway knowledge into gene selection algorithms were investigated: Mean expression profile of member genes within KEGG pathways (aveExpPath) [22], graph diffusion kernels for SVMs (graphK; diffusion kernel parameter δ=1) [12], p-step random walk kernels for SVMs (graphKp; parameters p=3, α=2, as suggested by Gao et al.) [23], pathway activity classification (PAC) [13], gradient boosting (PathBoost) [14] and network-based SVMs (parameter *sd*. *cutoff=0.8* for pre-filtering of probesets according to their standard deviation) [15]. In case of avgExpPath whole KEGG-pathways were selected or not selected based on their average differential expression between patient groups. This was done based on a SAM-test with FDR cutoff 5% (see above). In case of diffusion and p-step random walk kernels the SVM-RFE algorithm was adopted for gene selection using the implementation in the R-package pathClass [24]. Furthermore, pathClass was used to calculate the diffusion kernel. This implementation is directly based on [12] and only keeps the 20% smallest eigenvalues and corresponding eigenvectors of the normalized graph Laplacian to compute the kernel matrix.

PAC and PathBoost come with an own mechanism to select relevant genes. PathBoost incorporates network knowledge directly into the gradient boosting procedure to perform gene selection, whereas PAC first selects genes within each KEGG-pathway based on a *t*-test and then summarizes gene expression in each pathway to a pathway activity score. According to the original paper by Lee et al. [13] only the top 10% pathways with highest differences in their activity between sample groups were selected. Recently, Taylor et al. [16] found that differentially expressed hub proteins in a protein-protein interaction network could be related to breast cancer disease outcome. We here applied their approach (called HubClassify) as follows: the random permutation test proposed in Taylor et al. [16] was used to select differentially expressed hub genes with FDR cutoff 5%. Hubs were here defined to be those genes, whose node degree fell into the top 5% percentile of the degree distribution of our protein interaction network. Afterwards a SVM was trained using only those differential hub genes. Finally, we considered the recently proposed Reweighted Recursive Feature Elimination (RRFE) algorithm [17], which combines GeneRank [25] and SVM-RFE as implemented in the pathClass package [24]. In summary average pathway expression (aveExpPath), graph diffusion kernels for SVMs (graphK), p-step random walk graph kernels for SVMs (graphKp), PAC, PathBoost, networkSVM and HubClassify are considered in our comparison of network-based gene selection methods.

For all SVM classifiers used in this study the soft-margin parameter *C* was tuned in the range 10^-3^, 10^-3^, 10^-2^, ..., 10^3^ on the training data. For that purpose the pathClass package was employed, which uses the span-bound for SVMs as a computationally attractive and probably accurate alternative to cross-validation [26]. For elastic net SVMs and SCAD-SVMs we used the R-package penalizedSVM [27], which allows for tuning of hyperparameters (elastic net: λ_1_∈**[**2^-8^,2^14^**]**, λ_2_ set in a fixed ratio to λ_1_ according to [8]; SCAD-SVM: λ∈**[**2^-8^,2^14^**]**) based on the generalized approximate cross-validation (GACV) error as another computationally attractive alternative to cross-validation. The EPSGO algorithm described in [28] was used for finding optimal hyper-parameter values within the defined ranges. Note that in any case only the training data were used for hyper-parameter tuning.

It should be mentioned that for conventional approaches all probesets on the chip were considered. This is in agreement with a typical purely data driven approach with no extra side information. Please note that an a-priori restriction to probesets, which can be mapped to a pre-defined network, would already include a certain level of extra background knowledge with corresponding assumptions.

### **Classification performance and stability of a signature**

In order to assess the prediction performance for our tested gene selection methods, we performed a 10 times repeated 10-fold cross-validation. That means the whole data was randomly split into 10 folds, and each fold sequentially left out once for testing, while the rest of the data was used for training and optimizing the classifier (including gene selection via filtering methods, standardization of expression values for each gene to mean 0 and standard deviation 1, etc.). The whole process was repeated 10 times. It should be noted extra that also standardization of gene expression data was only done on each training set separately and the corresponding scaling parameters then applied to the test data.

The area under receiver operator characteristic curve (AUC) [29] was used here to measure the prediction accuracy, and the AUC was calculated by R-package ROCR [30]. To assess the stability of features selection methods, we computed the selection frequency of each gene within the 10 times repeated 10-fold cross-validation procedure. That means a particular gene could be selected at most 100 times.

### **Functional analysis of signature genes**

To interpret a signature gene in terms of biological function, we performed an enrichment analysis in terms of cancer-related disease genes, KEGG pathways and known drug targets for the prognosis biomarkers via Fishers exact test. We employed FunDO [31] to look for enrichment of disease related genes. FunDO uses a hyper-geometric test to find relevant diseases. Multiple testing correction was done using Bonferronis method [32]. Furthermore, an analysis of enriched KEGG pathways based on a hypergeometric test was done (multiple testing correction via Benjamini-Yekutieli’s method [33]). We also carried out an enrichment analysis for known targets of therapeutic compounds against breast cancer. For that purpose, we retrieved a list of 104 proteins and respective therapeutic compounds in breast cancer, which are either in clinical trials (also withdrawn ones), FDA approved or on the market with the help of the software MetaCore™ (see Additional file 1: Table S1). Fisher’s exact test was then used to assess statistical overrepresentation of drug targets within each signature.

### **Datasets**

#### ***Microarray gene expression data***

We collected six public breast cancer Affymetrix HGU133A microarray (22,283 probesets) datasets [34-39], which are further described in Table 1. The six datasets were obtained via Gene Expression Omnibus [40], and normalization was carried out using FARMS [41]. As clinical end points we considered metastasis free (datasets by Schmidt et al., Ivshina et al.) and relapse free (other datasets) survival time after initial clinical treatment, depending on the availability of the corresponding information in the original data. Time information was dichotomized into two classes according whether or not patients suffered from a reported relapse/metastasis event within 5 years. Patients with a survival time shorter than 5 years without any reported event were not considered and removed from our datasets.

**Table 1:** Employed breast cancer data sets

**GEOid**	**Patients**	**dmfs/rfs with event < 5 years**	**dmfs/rfs _ 5 years**	**source**
GSE2034	286	93	183	Wang et al. 2005 [34]
GSE1456	159	34	119	Pawitan et al. 2005 [35]
GSE2990	187	42	116	Sotiriou et al. 2006 [36]
GSE4922	249	69	159	Ivshina et al. 2006 [37]
GSE7390	198	56	135	Desmedt et al. 2007 [38]
GSE11121	200	28	154	Schmidt et al. 2008 [39]

#### ***Protein-protein interaction (PPI) network***

A protein interaction network was compiled from a merger of all non-metabolic KEGG pathways [42]- only gene-gene interactions were considered – together with the Pathway Commons database [43], which was ownloaded in tab-delimited format (May 2010). The purpose was to obtain an as much as possible comprehensive network of known protein interactions.

For the Pathway Commons database the *SIF* interactions INTERACTS_WITH and STATE_CHANGE were taken into account^a^ and any self loops removed. For retrieval and merger of KEGG pathways, we employed the R-package KEGGgraph [44]. In the resulting undirected network graph we had 13,840 nodes and 397,454 edges. Nodes in this network were identified via Entrez gene IDs.

The R package, *hgu133a.db*[45], was employed to map probe sets on the microarray to nodes in the PPI-network. This resulted in a protein-protein interaction network matrix of dimension 8886×8886, because several probe sets can map to the same protein in the PPI-network. Accordingly, expression values for probesets on the microarray that mapped to the same gene in the network were averaged. Probesets, which could not be mapped to the PPI network, were ignored for all network based approaches except for RRFE, which according to Johannes et al. [17], assigns a minimal gene rank to them.

## Results and discussion

### **Predictive power and stability**

**Figure 1:**
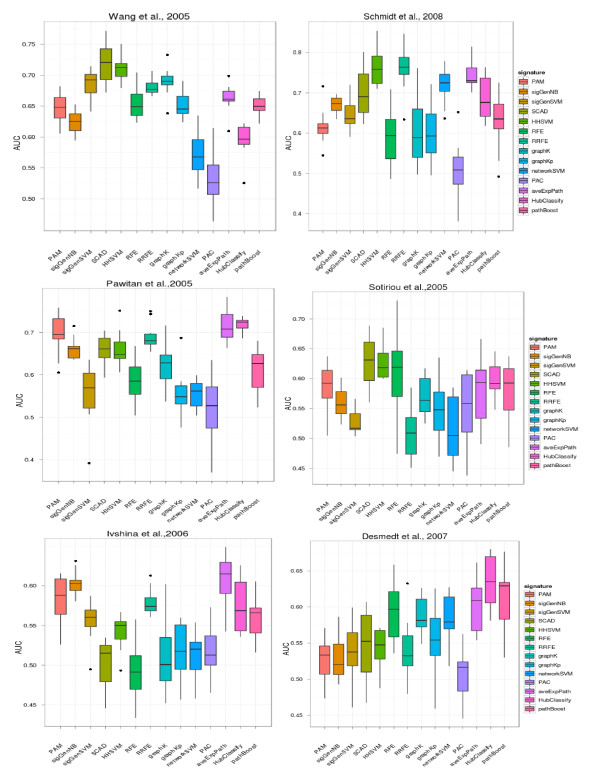
**Prediction performance in terms of area under ROC curve (AUC) PAM (prediction analysis of microarray data)**, *sigGenNB* (SAM + Naïve Bayes), *sigGenSVM* (SAM + SVM),*SCADSVM*, *HHSVM* (Huberized Hinge loss SVM), *RFE* (Recursive Feature Elimination), *RRFE* (Reweighted Recursive Feature Elimination), *graphK* (graph diffusion kernels for SVMs), *graphKp* (p-step random walk graph kernel for SVMs), *networkSVM* (Network-based SVM), *PAC* (Pathway Activity Classification), *aveExpPath* (average pathway expression), *HubClassify* (classification by significant hub genes), pathBoost.

**Figure 2:**
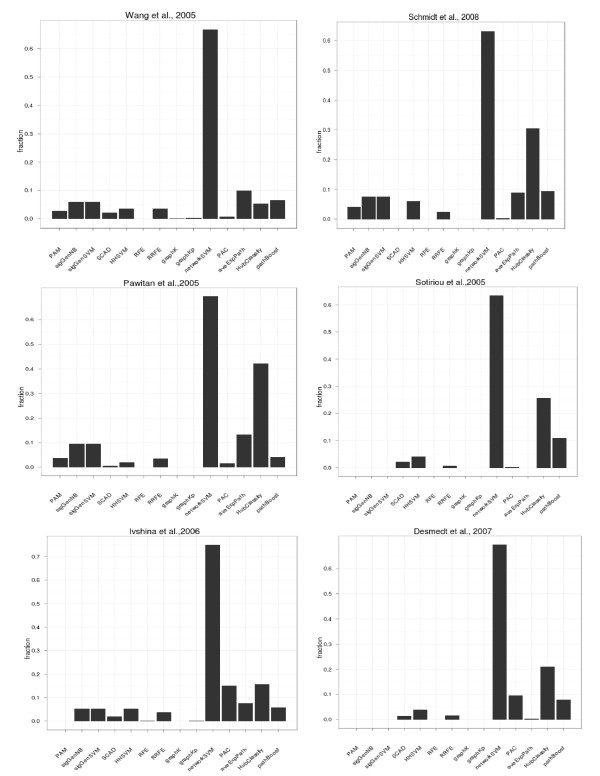
**Signature stability.** The y-axis shows the fraction of genes, being selected between 91 and 100 times.

We assessed the prediction performance of prognostic biomarker gene signatures obtained by fourteen gene selection methods on six gene expression datasets in terms of area under ROC curve (AUC) (Figure 1). The gene selection stability of each gene selection method is depicted in Figure 2 (fraction of constantly selected probe sets) and Additional file 2: Figure S1 in the Supplements (fraction of probe set that were selected 10, 20, …, 100 times during the 10 x 10-fold CV procedure).

In general, we observed a large variability of prediction performances of individual methods between different datasets. This is not necessarily surprising, since it is known that the performance of any machine learning algorithms is dependent on the data at hand. Moreover, each dataset under study here contains different patients with unique characteristics and also clinical end points were slightly different (relapse free versus metastasis free survival after treatment). We are convinced that a comparison on a larger number of datasets reveals more of the true variability of an algorithm than a typical comparison on few selected ones.

We combined median AUC values for all methods across datasets into a summary plot (Additional file 3: Figure S2) and assessed the statistical significance between methods via a 2-way ANOVA analysis with Tukey’s post-hoc test. Briefly, the ANOVA analysis modelled AUC values by a method and a dataset factor as well as an interaction term between them. This generally revealed only small effect sizes (average differences between methods), of which, nonetheless, a couple could be identified as statistically significant (Additional file 4: Table S2, Additional file 5: Table S3, Bonferroni adjusted *p*-value cutoff 5%). Overall PAC, graph diffusion kernels, p-step random walk kernels, RFE and significant gene SVMs were almost consistently outperformed by the other methods. On the other hand, HHSVM and average pathway expression were identified as best performing methods (Table 2). A general advantage for network based approaches could not be identified among our tested methods. However, some network-based methods (specifically network-based SVM, hub-based classification, pathBoost) revealed significantly higher gene selection stability (Figures 2, Additional file 2: Figure S1). Network-based SVMs performed clearly outstanding here. The reason might be two-fold: On one hand network-based SVMs come with a pre-filtering step of probesets according to their standard deviation, which already drastically reduces the set of considered probesets for the later learning phase and thus naturally enhances stability. On the other hand network-based SVMs have a very effective mechanism for grouped selection of network connected genes via the infinity norm penalty [15]. Nonetheless, we found network-based SVMs to show a comparably poor prediction performance. This underlines that an improved gene selection stability does not necessarily coincide with better prediction performance. The reason for this behaviour could be that many genes reveal a high correlation in their expression. If such highly correlated genes are itself correlated with the patient group, then picking any of these genes leads to a similar prediction performance. On the other hand, picking preferentially one particular gene out of the correlated group (as tried by network-based approaches) increases gene selection stability, but does not necessarily increase prediction performance, either. This is exactly the behaviour we can observe in our datasets: Some network-based approaches (specifically networkSVM) have significantly improved gene selection stability, but do not perform consistently better than conventional methods, like PAM. We would like to point out that the high stability of network based SVMs and hub based classification is not at all associated to a higher number of selected genes (Additional file 6: Figure S3).

**Table 2:** ANOVA analysis for prediction performance (AUC)

**gene selection method**	**no. significant wins**	**network based**
PAM	4	No
sigGenNB	3	No
sigGenSVM	2	No
SCAD	6	No
**HHSVM**	**9**	No
RFE	1	No
RRFE	6	Yes
graphK	2	Yes
graphkKp	1	Yes
networkSVM	1	Yes
PAC	0	Yes
**aveExpPath**	**9**	Yes
HubClassify	6	Yes
pathBoost	4	Yes
**network based (average)**	**3.625**	
**classical (average)**	**4.17**	

*PAM* (prediction analysis of microarray data), *sigGenNB* (SAM + Naïve Bayes), *sigGenSVM* (SAM + SVM),*SCAD-SVM*, *HHSVM* (Huberized Hinge loss SVM), *RFE* (Recursive Feature Elimination), *RRFE* (Reweighted Recursive Feature Elimination), *graphK* (graph diffusion kernels for SVMs), *graphKp* (p-step random walk graph kernel for SVMs), *networkSVM* (Network-based SVM), *PAC* (Pathway Activity Classification), *aveExpPath* (average pathway expression), *HubClassify* (classification by significant hub genes), pathBoost.

We went on to investigate gene selection stability in more depth. For that purpose we introduced a gene selection stability index (*SI*) for each algorithm across datasets: For each algorithm we recorded the fraction of genes being selected 1–10, 11–20, 21–30, 31–40, 41–50, 51–60, 61–70, 71–80, 81–90, 91–100 times on dataset *i*. These fractions (basically making up a histogram) are summarized into a vector fi. The theoretically optimal gene selection behavior would be the vector ***e*** = (0,0,0,0,0,0,0,0,0,1)^*T*^, which means that all genes are selected consistently. Based on that we computed a gene selection stability index (*SI*) for each algorithm across datasets:

(1) $$SI=\frac{1}{n}\sum_{i-1}^{n}{\left\|||,f_{i}-e||\right\|}_{2}^{2}$$

Here *n* is the number of datasets. A ranking of all algorithms according to this stability index is shown in Table 3. This highlighted the much different behavior of networkSVM compared to all other approaches, which, given our previously discussed findings, was not very surprising. As second best method with respect to gene selection stability we identified hub-based classification. The high stability of this approach can be explained by the a-priori restriction on hub genes.

**Table 3:** Gene selection stability according to stability index (lower = better)

**gene selection method**	**GSE2034**	**GSE11121**	**GSE1456**	**GSE2990**	**GSE4922**	**GSE7390**	**Median**
PAM	0.237	0.282	0.259	0.302	0.281	0.277	0.279
sigGenNB	0.209	0.193	0.173	0.289	0.208	0.272	0.208
sigGenSVM	0.209	0.193	0.173	0.289	0.208	0.272	0.208
SCAD	0.245	0.265	0.268	0.232	0.229	0.251	0.247
HHSVM	0.212	0.191	0.210	0.199	0.197	0.205	0.202
RFE	0.285	0.298	0.295	0.287	0.293	0.291	0.292
RRFE	0.224	0.240	0.211	0.246	0.209	0.248	0.232
graphK	0.276	0.290	0.295	0.285	0.283	0.285	0.285
graphkKp	0.269	0.281	0.276	0.271	0.273	0.276	0.274
networkSVM	**0.021**	**0.027**	**0.018**	**0.026**	**0.014**	**0.018**	**0.020**
PAC	0.249	0.257	0.245	0.259	0.158	0.181	0.248
aveExpPath	0.189	0.192	0.156	0.294	0.190	0.237	0.191
HubClassify	0.215	**0.095**	**0.073**	0.106	0.138	0.120	0.113
pathBoost	0.200	0.206	0.247	0.199	0.235	0.213	0.210

We also investigated gene selection from a different perspective. We ran a SAM analysis on each of our datasets and plotted the fraction of top the 100 genes (sorted by FDR) that were selected by each investigated algorithm (Additional file 7: Figure S4). This analysis thus focused on the ability of algorithms to select differentially expressed genes. Not very surprisingly, in this comparison significant gene SVM and NB revealed the top performance, followed by average pathway expression. This shows that selected pathways typically contained many genes with high fold change, which altered the overall average pathway activity together.

### **Biological interpretability**

To investigate the biological interpretability of our found signatures, we performed an enrichment analysis with respect to KEGG pathways, Disease Ontology terms and known drug targets. For that purpose we trained each of the above described methods once on the whole dataset to retrieve a final gene signature.

**Figure 3:**
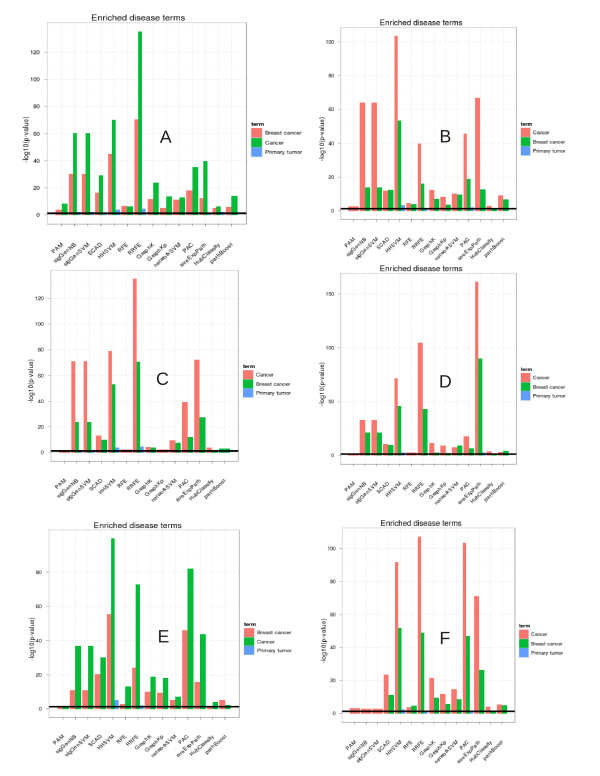
**Interpretability of signatures (enriched disease genes).** For aveExpPath and PAC the enrichment of the particular disease category within selected pathway genes is shown. **A** represents data GSE2034 [34]; **B** represents data GSE11121 [39]; **C** represents data GSE1456 [35]; **D** represents data GSE2990 [36]; **E** represents data GSE4922 [37]; **F** represents data GSE7390 [38].

**Figure 4:**
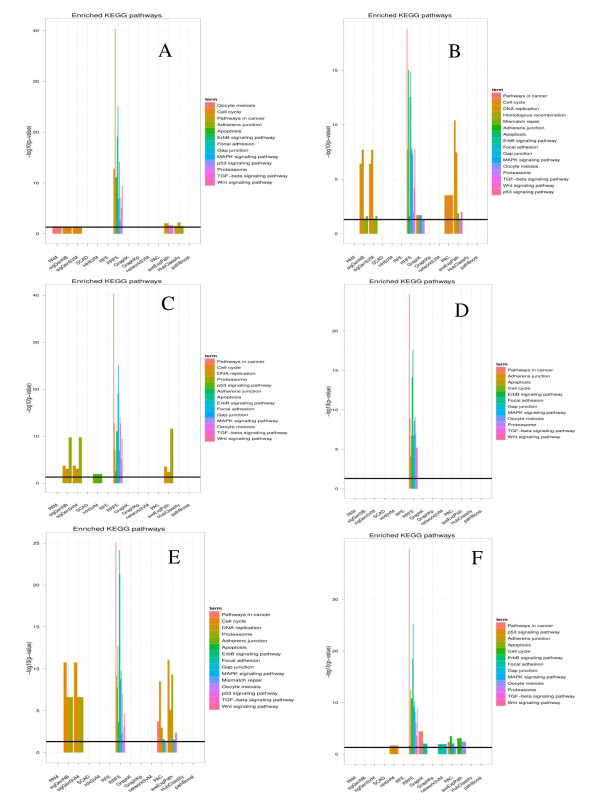
**Interpretability of signatures (enriched KEGG-pathways).** For aveExpPath the adjusted p-value for differential expression from the SAM-test is shown. For all other methods we tested pathway enrichment within the set of selected genes.

**Figure 5:**
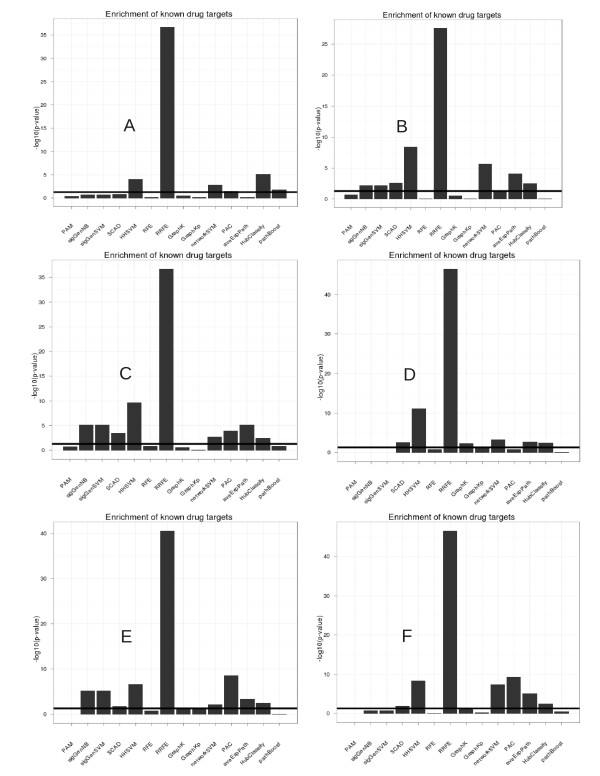
**Interpretability of signatures (enriched drug targets).** For aveExpPath and PAC the enrichment of drug targets within selected pathway genes is shown.

In generally, this analysis revealed a high enrichment of disease related genes, KEGG pathways and known drug targets in signatures selected by network-based approaches (Figures 3,4,5). Specifically, RRFE (and partially also aveExpPath with regard to pathways) yielded an extremely high enrichment with respect to all three categories on all datasets. The overrepresentation of known drug targets for genes selected by RRFE was absolutely outstanding on all datasets. Consistently enriched KEGG-pathways for gene signatures selected by RRFE and aveExpPath were Pathways in cancer, MAPK signaling pathway, ErbB signaling pathway, Adherens junction and Focal adhesion, which have all been related to breast cancer [46-49].

The reason for the good interpretability of pathways selected by AvgExpPath is directly clear, since this method focuses on selection of whole pathways. The outstanding interpretability of genes selected by RRFE can be explained as follows: RRFE uses a modification of Google’s PageRank algorithm (GeneRank – [25]) to compute for each gene a rank according to its own fold change and its connectivity with many other differentially expressed ones (guilt by association principle). This rank is then used to re-scale the hyperplane normal vector of a SVM. This method automatically leads to a preference of genes which are central in the network (c.f. [17]). These central genes are often well studied and directly known to be disease related [50].

## Conclusion

In this paper we performed a comprehensive and detailed comparison of fourteen gene selection methods (eight integrating network information) in terms of prediction performance, gene selection stability and interpretability on six public breast cancer datasets.

In general we could not identify one single algorithm to perform best with respect to all three categories. Much more, we found that incorporating network of pathway knowledge into gene selection methods in general did not significantly improve classification accuracy compared to classical algorithms. Network-based SVMs drastically enhanced gene selection stability, but showed a comparably poor prediction performance. On the other hand RRFE lead to highly interpretable gene signatures with moderate prediction accuracy, but certainly not extremely high stability (although significantly better than RFE). Relatively simple gene selection methods, like average pathway expression, revealed a good prediction accuracy. Similar results have been reported in Haury et al. [51]. Nonetheless, it is worth mentioning that the crucial assumption made by average pathway expression, namely that the mean pathway activity is altered significantly between two patient groups, might not always be fulfilled, for instance, if only few genes in a pathway are differentially expressed. Thus this method should be applied with care.

We found HHSVM and SCAD-SVM in most cases to show a better prediction performance than SVM-RFE. This is, for instance, in agreement with [8] and [52], who explained that by the fact that elastic net and SCAD penalties can better deal with correlated features, which are typically observed in gene expression data. In our comparison HHSVM, together with average pathway expression, revealed the highest prediction performance.

It appears in our comparison that incorporation of biological network knowledge into gene selection methods does not necessarily help to improve the prediction accuracy of prognostic biomarkers. Integrating additional experimental data, such as microRNA measurements, SNP or CNV data in addition to protein-protein interaction information might offer an alternative route to enhance prediction performance as well as stability and interpretability of biomarker signatures in the future.

To our knowledge this paper is one of the most detailed and largest comparisons, which has been conducted so far to assess the performance of network-based gene selection methods in a multi-dimensional way. Whereas most previous approaches concentrated only on one aspect of gene selection methods, namely prediction performance, we have here also looked to stability and interpretability of the tested algorithms. Prognostic and diagnostic gene signatures are applied in a biomedical context. Thus, the classical machine learning based perspective of focusing only on prediction performance might be too narrow. Indeed we believe that stability and interpretability of gene signatures will strongly enhance their acceptance and practical applicability for personalized medicine. Here we see the largest potential for methods, which incorporate biological background knowledge, for example in form of pathway knowledge, known disease relations or other approaches. This does not, of course, imply that prediction performance should be sacrificed for reproducibility or interpretability, but seen as an additional goal to achieve.

## Endnotes

^a^http://www.pathwaycommons.org/pc/sif_interaction_ rules.do

## Competing interests

The authors declare that they have no competing interests.

## Authors’ contributions

YC conducted the programming work and executed the computational experiments. HF guided the project and gave general advises. Both authors contributed to writing the text, read and approved the finial manuscript.

## Supplementary Material

Additional file 1 **Table S1.** Known drug targets for breast neoplasms. Click here for file

Additional file 2 **Figure S1.** Stability of each gene selection methods. The y-axis shows the fraction of genes, being selected 1–10, 11–20, 21–30, 31–40, 41–50, 51–60, 61–70, 71–80, 81–90 and 91–100 times. PAM (prediction analysis of microarray data), sigGenNB (SAM + Naïve Bayes), sigGenSVM (SAM + SVM),SCAD-SVM, HHSVM (Huberized Hinge loss SVM), RFE (Recursive Feature Elimination), RRFE (Reweighted Recursive Feature Elimination), graphK (graph diffusion kernels for SVMs), graphKp (p-step random walk graph kernel for SVMs), networkSVM (Network-based SVM), PAC (Pathway Activity Classification), aveExp-Path (average pathway expression), HubClassify (classification by significant hub genes), pathBoost. Click here for file

Additional file 3 **Figure S2.** Median AUC values across all datasets. Click here for file

Additional file 4 **Table S2.** Tukey's post-hoc test analysis for AUC values (5% significance cutoff). Click here for file

Additional file 5 **Table S3.** Tukey's post-hoc test analysis for SI values (5% significance cutoff). Click here for file

Additional file 6 **Figure S3.** Number of selected genes per method. Click here for file

Additional file 7 **Figure S4.** Fraction of differentially expression genes in signatures. Click here for file
