# Supplementary figures and images for: Prognostic gene signatures for patient stratification in breast cancer - accuracy, stability and interpretability of gene selection approaches using prior knowledge on protein-protein interactions
Source: BMC Bioinformatics. 2012 May 1;13:69. doi: 10.1186/1471-2105-13-69 (PMC3436770; doi:10.1186/1471-2105-13-69)

Wang et al., 2005

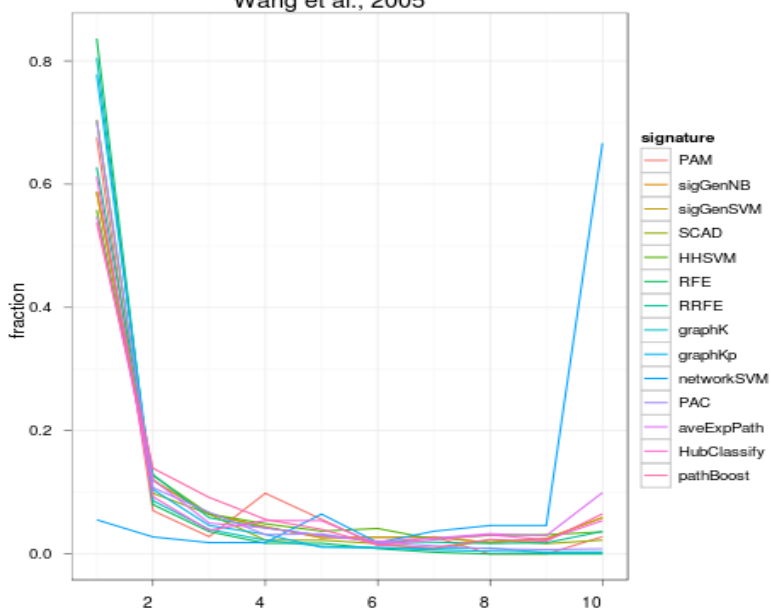

Schmidt et al., 2008

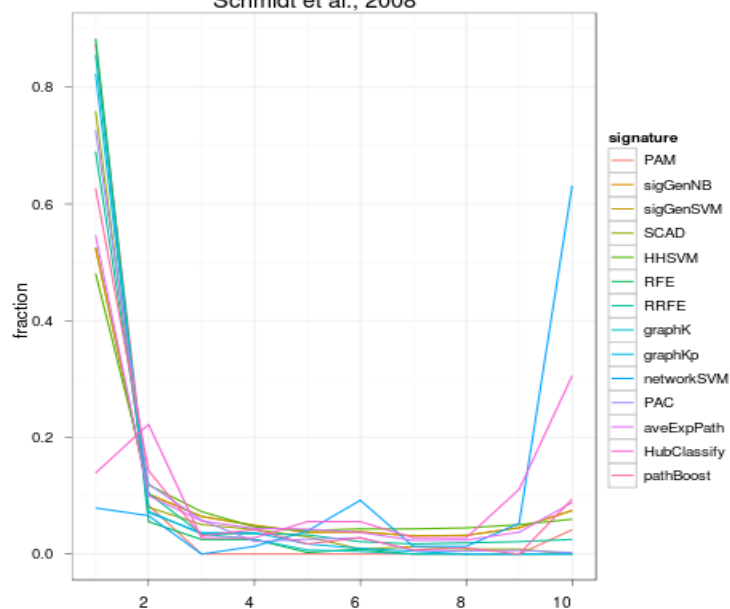

Pawitan et al., 2005

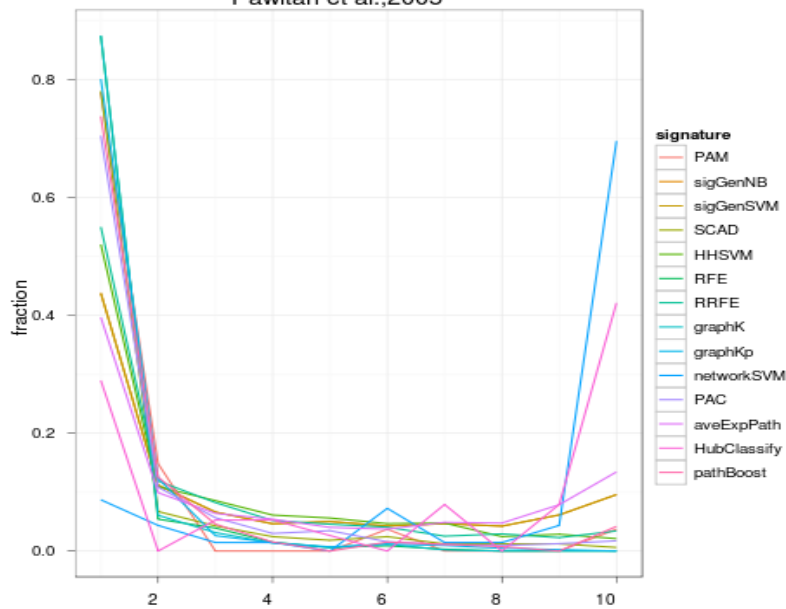

Sotiriou et al., 2005

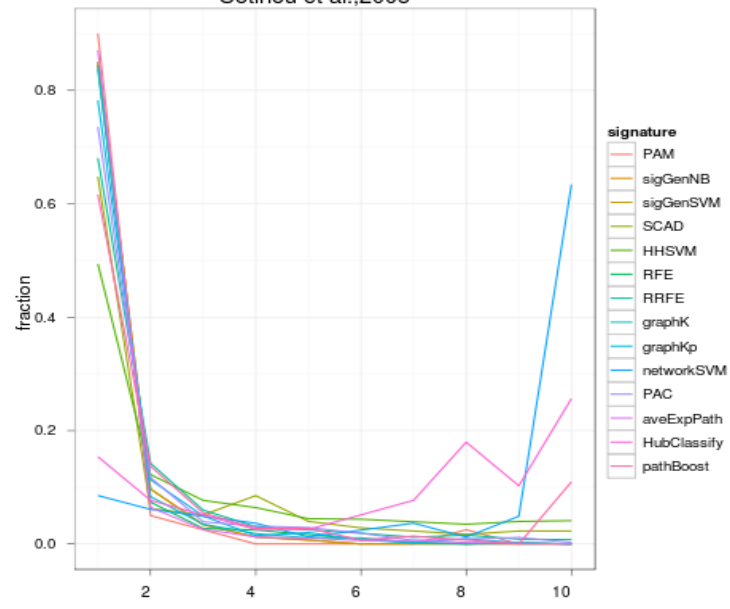

Ivshina et al., 2006

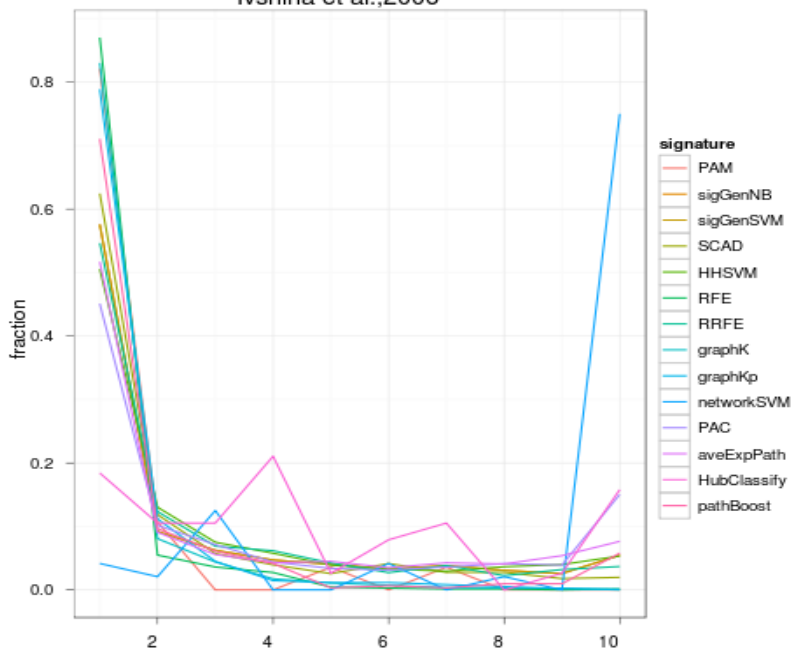

Desmedt et al., 2007

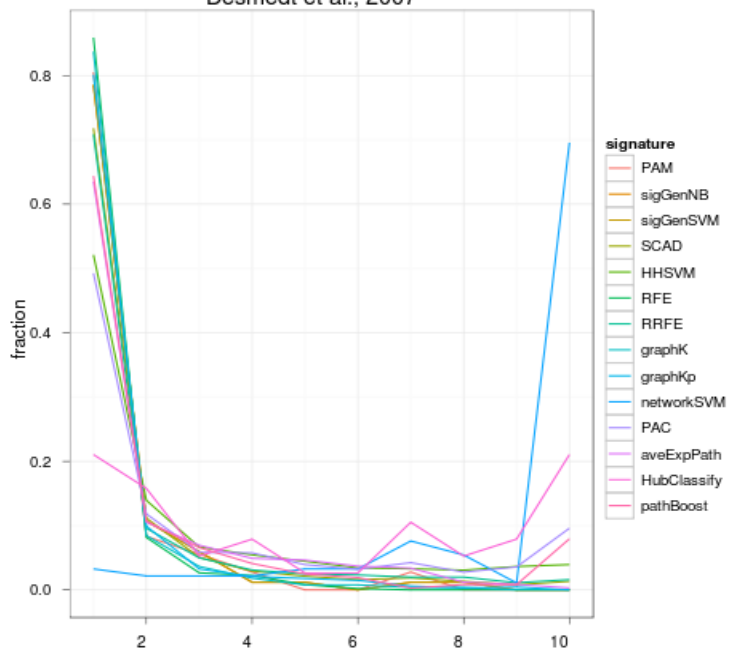

Supplement: Additional file 2 — Figure S1. Stability of each gene selection methods. The y-axis shows the fraction of genes, being selected 1–10, 11–20, 21–30, 31–40, 41–50, 51–60, 61–70, 71–80, 81–90 and 91–100 times. PAM (prediction analysis of microarray data), sigGenNB (SAM + Naïve Bayes), sigGenSVM (SAM + SVM),SCAD-SVM, HHSVM (Huberized Hinge loss SVM), RFE (Recursive Feature Elimination), RRFE (Reweighted Recursive Feature Elimination), graphK (graph diffusion kernels for SVMs), graphKp (p-step random walk graph kernel for SVMs), networkSVM (Network-based SVM), PAC (Pathway Activity Classification), aveExp-Path (average pathway expression), HubClassify (classification by significant hub genes), pathBoost. [file 1471-2105-13-69-S2.PDF]

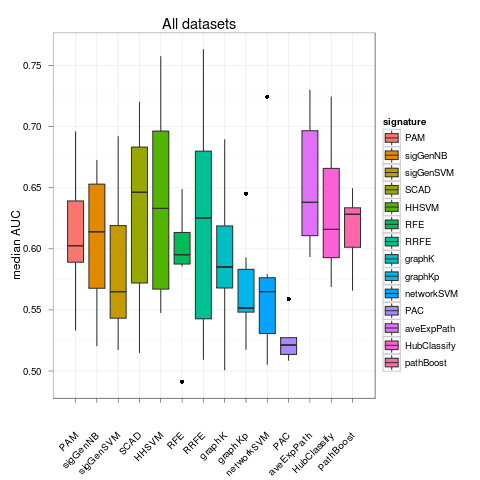

Supplement: Additional file 3 — Figure S2. Median AUC values across all datasets. [file 1471-2105-13-69-S3.PNG]

Wang et al., 2005

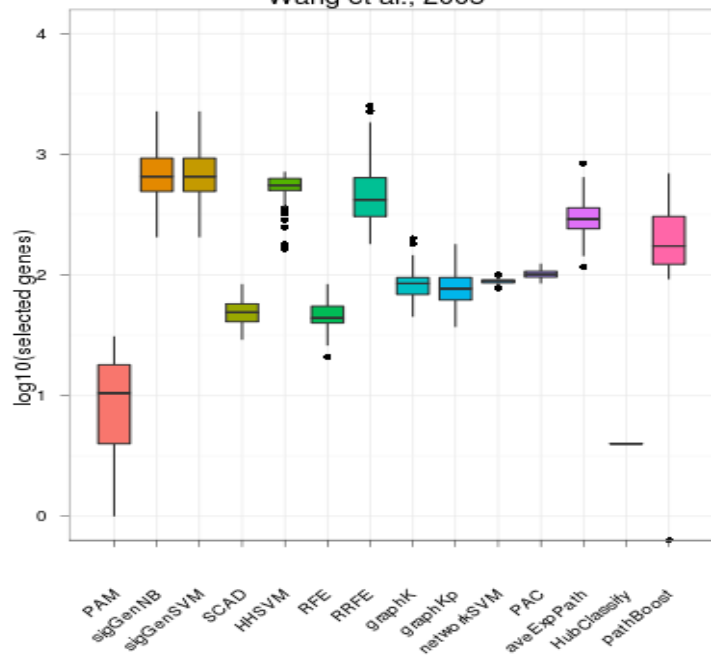

Schmidt et al., 2008

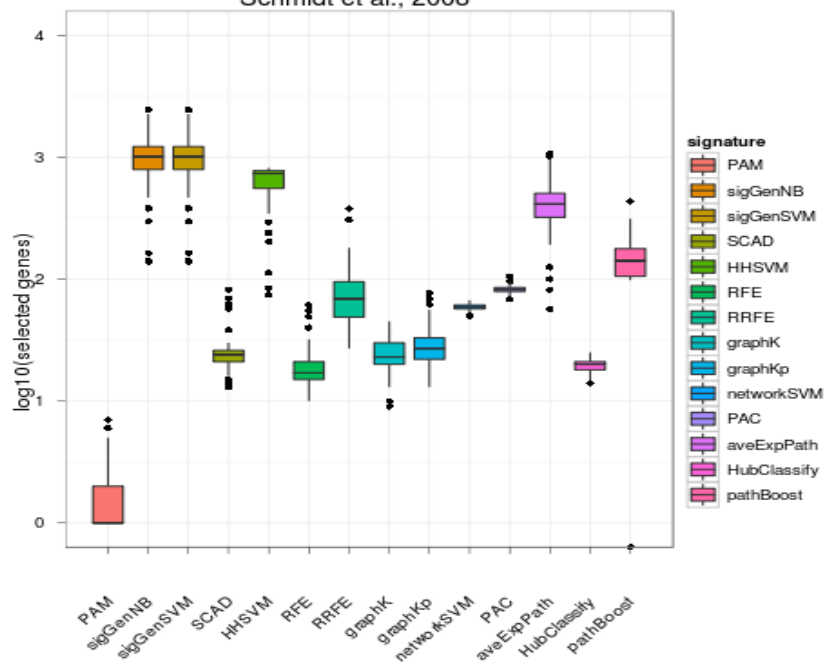

Pawitan et al., 2005

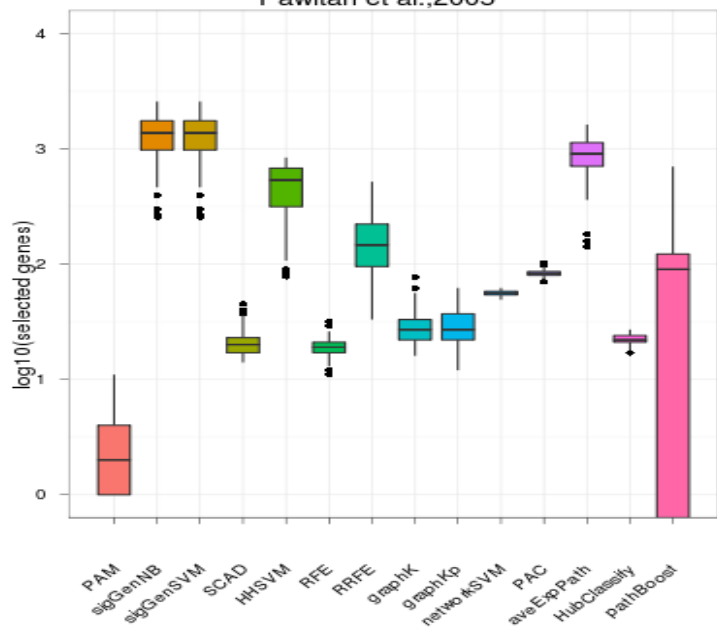

Sotiriou et al., 2005

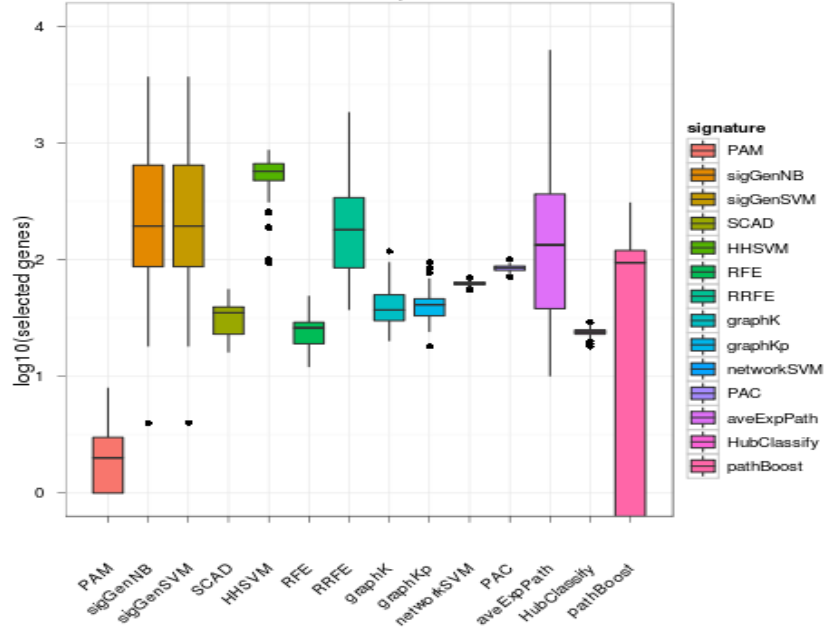

Ivshina et al., 2006

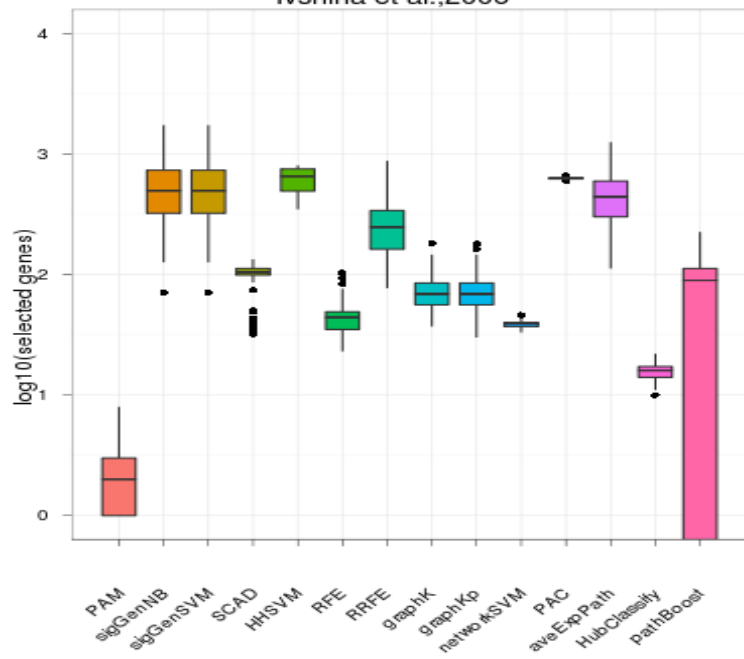

Desmedt et al., 2007

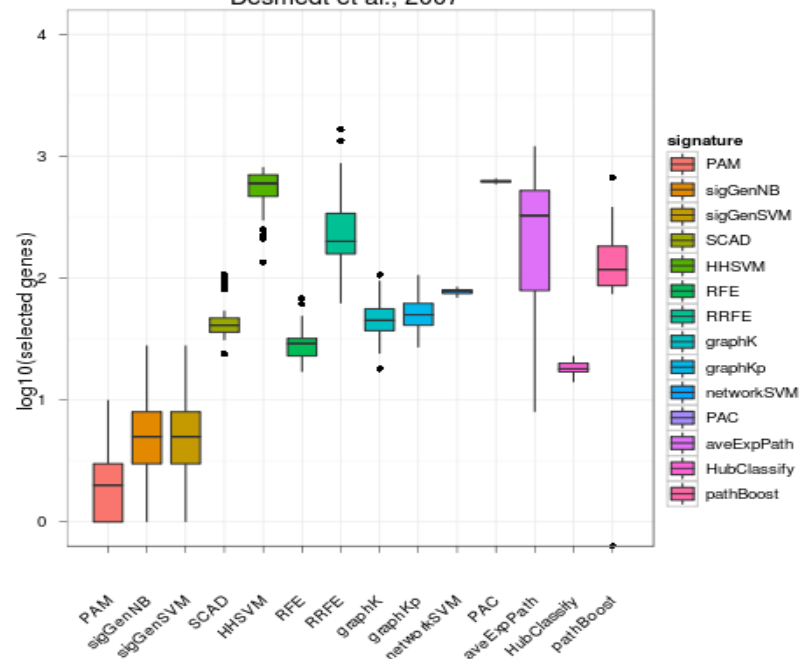

Supplement: Additional file 6 — Figure S3. Number of selected genes per method. [file 1471-2105-13-69-S6.PDF]

Wang et al., 2005

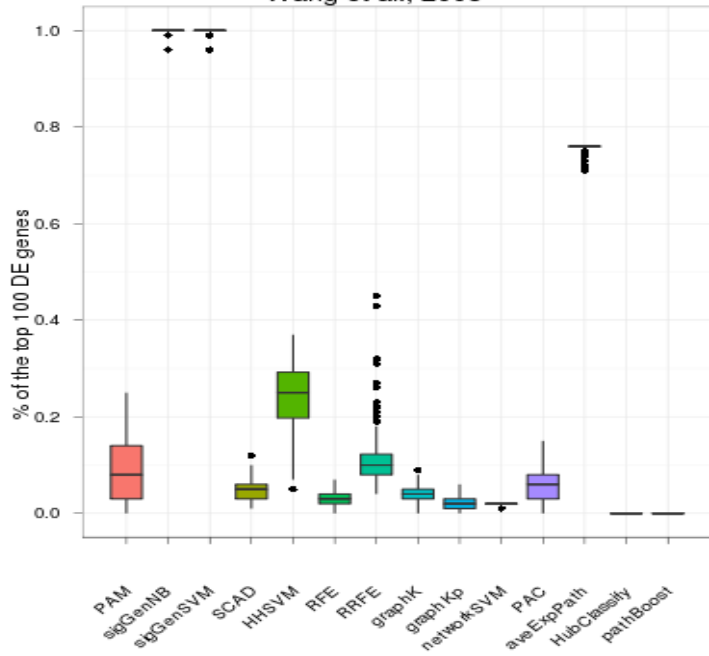

Schmidt et al., 2008

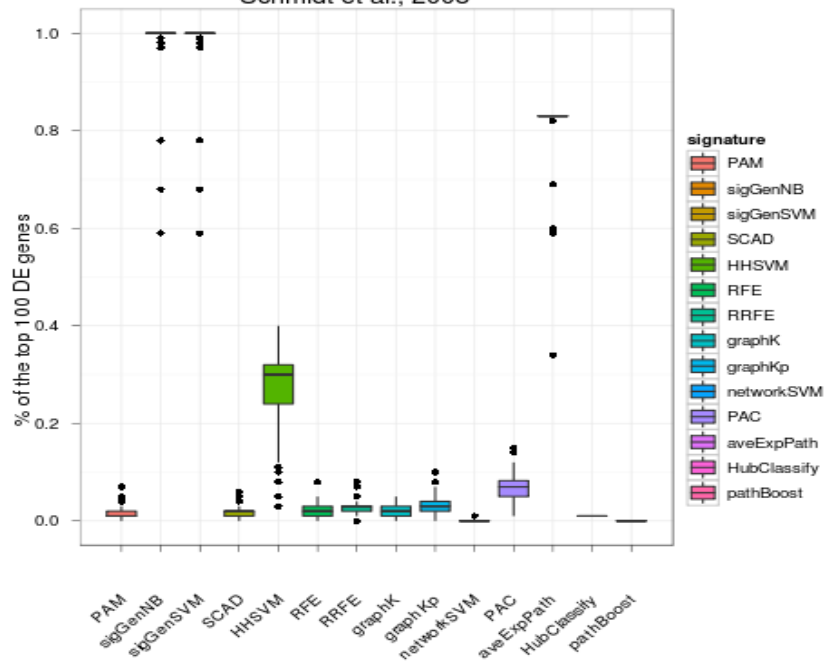

Pawitan et al., 2005

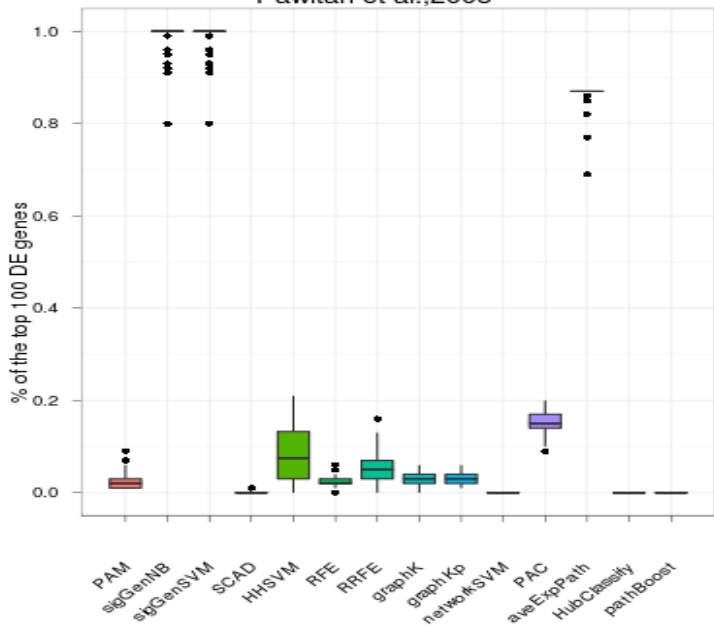

Sotiriou et al., 2005

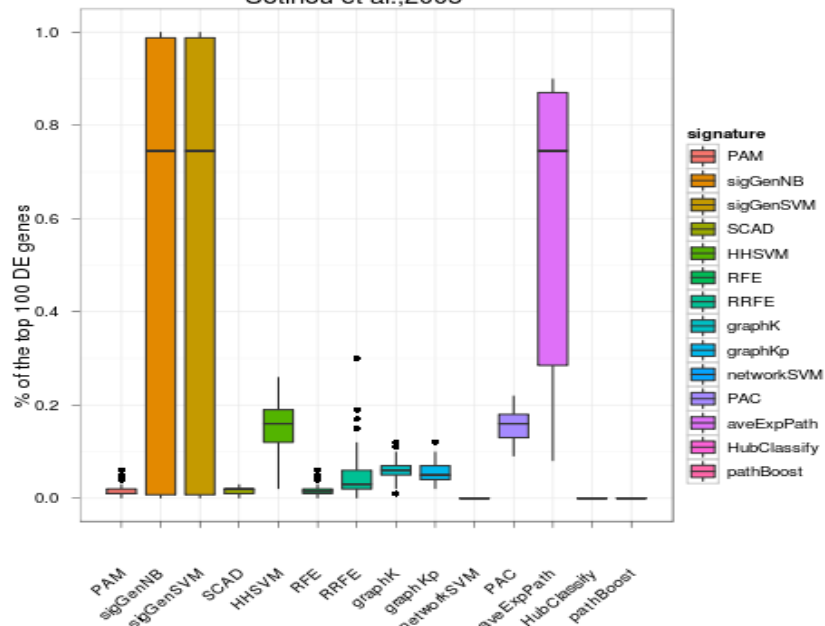

Ivshina et al., 2006

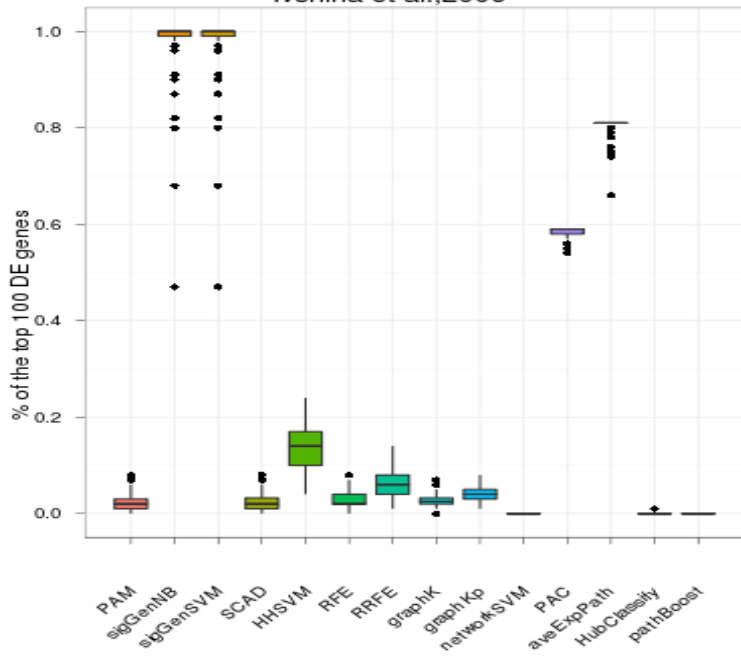

Desmedt et al., 2007

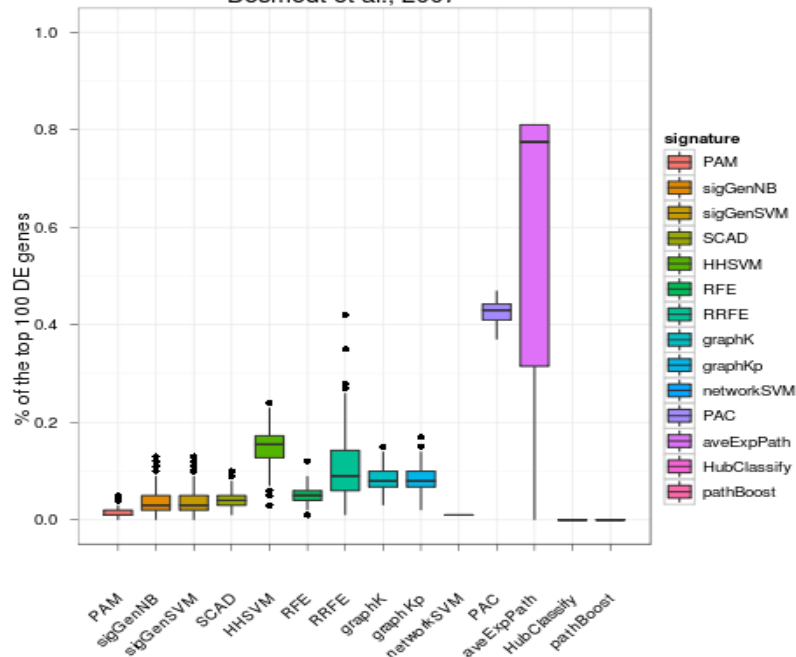

Supplement: Additional file 7 — Figure S4. Fraction of differentially expression genes in signatures. [file 1471-2105-13-69-S7.PDF]
